# Cryothermal Energy Ablation Of Cardiac Arrhythmias 2005: State Of The Art

**Published:** 2005-01-01

**Authors:** Roberto De Ponti

**Affiliations:** Department of Cardiovascular Sciences, Ospedale di Circolo e Fondazione Macchi, University of Insubria, Varese, Italy

**Keywords:** cryoablation, cardiac arrhythmia

## Abstract

At the time of antiarrhythmic surgery, cryothermal energy application by a hand-held probe was used to complement dissections and resections and permanently abolish the arrhythmogenic substrate. Over the last decade, significant engineering advances allowed percutaneous cryoablation based on catheters, apparently not very different from standard radiofrequency ablation catheters. Cryothermal energy has peculiar characteristics. In fact, it allows testing in a reversible way the effects of energy application at higher temperature, before producing a permanent lesion at -75°C. Moreover, slow formation of the lesion allows timely discontinuation of the application, as soon as inadvertent modifications of normal atrioventricular conduction are observed during ablation in the proximity of atrioventricular node and His bundle, avoiding its permanent damage. Over the last years, percutaneous cryothermal ablation has been widely used for a variety of cardiac arrhythmias. From the data gathered, it is unlikely that cryoablation will replace standard ablation in unselected cases. Nevertheless, for the above mentioned peculiarities, cryothermal ablation has proved very effective and safe for ablation of arrhythmogenic substrates close to the normal conduction pathways, becoming the first choice method to ablate anteroseptal and midseptal accessory pathways. It can be also the best treatment for ablation of the slow pathway to abolish atrioventricular node reentrant tachycardia in pediatrics or when particular anatomy of the Koch’s triangle is observed. Cryothermal ablation of the pulmonary veins for atrial fibrillation, although longer than radiofrequency ablation, is not associated with pulmonary vein stenosis and is expected to be less thrombogenic; new catheter designs for cryothermal ablation of this challenging arrhythmia are to be tested to assess their efficacy and clinical usefulness.

## From surgical to catheter-based cryoablation

In the 80’s, epicardial cryoablation was introduced in antiarrhythmic surgery for ablation of accessory pathways by Klein et al  [1]. A hand-held cryoprobe was applied at the site where intraoperative mapping localized the arrhythmogenic substrate. The probe was refrigerated to -60°C and its effectiveness was evaluated during continuous monitoring of the cardiac electrical activity. This method, largely used in the past [2-4] for antiarrhythmic surgery and still in use for the treatment of some forms of ventricular tachycardia [5,6], is safe and effective. During surgery, cryomapping allowed precise localization of the arrhythmogenic substrate by monitoring the effect of a cryothermal energy application with higher temperature (0 to -15°C) for a limited time (15-30s), before producing a permanent lesion at -65°C only in the most appropriate site [7].

**Figure 1:**
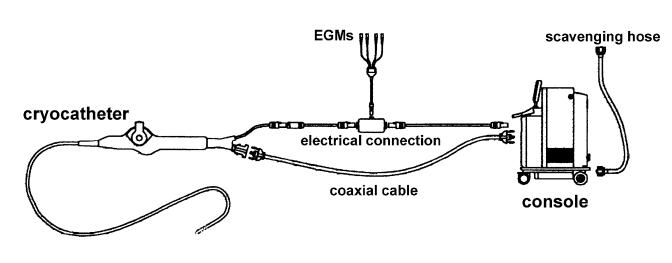
Scheme of cryoablation system. The steerable catheter and the console are connected by: 1) a coaxial cable, used both to deliver fluid nitrous oxide to the catheter and to remove separately the gas from the catheter; 2) electrical cable, which is connected both to the conventional recording system for electrograms (EGMs) analysis and storage and to the console for reading of the tip temperature. A tank of fluid nitrous oxide is located inside the console; the gas removed from the catheter to the console is evacuated through a scavenging hose into the vacuum line of the electrophysiology laboratory. The system has several sensors to avoid inadvertent leaks of nitrous oxide into the patient body and to check connections of the different cables to the console.

In the 90’s, significant engineering advances allowed the development of systems for percutaneous cryoablation, consisting of a steerable catheter, apparently not very different from standard ablation catheters for radiofrequency energy delivery, and a dedicated console (Figure 1). Fluid nitrous oxide is delivered under pressure to the catheter tip through a hollow injection tube, which runs internally for the whole length of the catheter. In a small chamber inside the tip electrode, nitrous oxide is made expand and a liquid to gas phase change takes place with heat extraction from the electrode-to tissue interface. The gas is constantly removed through a second coaxial lumen inside the catheter, under vacuum. The tip temperature is constantly monitored by the console, which in turn adjust the nitrous oxide flow to obtain and maintain the preset temperature. Two systems for cryoablation are currently available. The first is provided by Cryocath Technologies Inc. (Montreal, Canada) and utilizes 7 or 9 F steerable catheters with 4, 6 or 8 mm long tip electrode. The ablation catheter is connected to a dedicated console, which has two algorithms available: 1) for cryomapping with slow decrease of the temperature to - 30°C up for 80 s; 2) for cryoablation with faster decrease of the temperature to -75°C for up to 480 s. In any case, the target temperature can be manually preset on the console at any value between -30 and -75°C. The second system (CryoCor Inc., San Diego, California, USA) has 10 F steerable catheters with 6.5 or 10 mm long tip electrodes. The console has a built-in closed loop pre-cooler for the fluid nitrous oxide, whose flow at the catheter tip is adjusted during the application to maintain a temperature of -80°C.

## Lesion formation by cryothermal energy

Since cryothermal energy has been widely used in surgery, the types of cellular lesion caused by tissue freezing are well known [8]. The mechanisms underlying lesion formation by cryoenergy are two-fold: 1) a direct cell injury and 2) a vascular mediated tissue injury.

The direct cellular injury is due to ice formation, which has different distribution according to temperature reached during cooling. By cooling to mild temperature (0 to -20°C), ice forms only extracellularly. Consequently, extracellular environment becomes hyperosmotic and an intracellular to extracellular water shift occurs. This causes cellular shrinkage and damage to membrane. Cooling to these temperature may result in cellular death, if the application is enough prolonged. Using short applications with limited temperature, the effect produced on the cell is reversible and cellular function recovers, although minimal cellular damage may be produced. In the clinical use, the option of producing a functionally reversible lesion is quite attractive to test the effect of cryoablation without producing a permanent lesion. Conversely, by cooling down to -40°C and further, intracellular water freezes and formation of intracellular ice results in major and irreversible disruption of organelles and cell membrane with cellular death. Intracellular ice may propagate from one cell to another via intercellular channels.

The second mechanism underlying lesion formation by cryothermal energy delivery is a vascular-mediated mechanism. In fact, the initial tissue response to cooling is vasoconstriction with decreased blood flow. As tissue freezes, circulation ceases uniformly in the frozen tissue. The uniformity of cell death in a lesion produced by cryothermal energy has suggested ischemic necrosis as the main mechanism for tissue death, although it is impossible to distinguish the tissue damage caused by this mechanism from the one produced by intracellular ice formation. Upon re-warming, a hyperemic response is observed with increased vascular permeability and edema formation. Other than producing increased permeability and edema, endothelial damage results in platelet aggregation and micro-thrombus formation, with stagnation of microcirculation in about 30-45 min.

Especially in the percutaneous closed chest cryoablation, the effect produced by energy application is the result of a temperature gradient occurring at the electrode/tissue interface and possibly influenced by different factors, such as contact or blood flow. At the interface, the coldest area is the one adjacent to the catheter tip, where functional effects of energy delivery are observed earlier. Conversely, the less cooled area is the one at the periphery of the cryolesion, whose dimensions may also vary according to the duration of freezing. Due to limited (both in time and temperature) cooling of outer limit of the lesion, reversible tissue damage is more likely to occur in this area. As a consequence, the effects obtained late during cryothermal energy application are likely to revert early upon re-warming and, therefore, any expected functional modification induced by cryoenergy should occur early (usually within the first 30 s of the application) in order to obtain a successful and permanent ablation of a given arrhythmogenic substrate.

## Cryothermal vs radiofrequency energy: differences and their clinical implications

In closed chest cryoablation, the effect of cryothermal energy application greatly depends on the minimum temperature reached, the application duration and the temperature time constant [9]. The latter value indicates the course of the descent of temperature to the target temperature and a shorter value (expressed in seconds) identifies a more effective application. Due to intrinsic characteristics of cryothermal energy at a fixed minimum temperature, the lesion forms more slowly than the one produced by hyperthermic injury. This has two practical implications. The first is that the application duration for cryoablation is significantly longer than for radiofrequency energy and a lesion produced by cryothermal energy by a 4 mm tip 7 F cryocatheter at -75°C for 240 s has a comparable depth to the one obtained by radiofrequency energy applied in temperature control mode at 50 W, +70°C for 60 s [10]. Second, the longer estimated time required to create a permanent lesion may be clinically useful to better modulate the lesion formation in critical areas (i.e. close to the atrioventricular node-His bundle). In these cases, if inadvertent modifications of conduction over the normal pathways is observed during the application, immediate discontinuation of cryothermal energy application results in return to baseline conduction properties, with no permanent damage to normal conduction. Recently, it has been demonstrated in a canine model [10] that cryolesions are associated with significantly less endothelial disruption and overlying thrombus formation as compared to lesion produced by radiofrequency energy, regardless of the preventive use of aspirin. This characteristic could be very important, especially when multiple and prolonged energy applications are required to treat atrial arrhythmias in the left atrium. Unlike lesions produced by a hyperthermic injury, cryolesions show both in open chest [11] and closed chest [10,12] models, a well demarcated border zone and preservation of the extracellular collagen matrix with no collagen denaturation, nor contracture related to hyperthermic effects. These histologic observations combine with the clinical evidence that cryothermal energy application adjacent to coronary arteries, as well as in venous vessels (coronary sinus, middle cardiac vein and pulmonary veins) [12-15], does not results in damage nor chronic stenosis of their lumen.

In the already mentioned study [10], it has been pointed out that lesions by radiofrequency energy have a comparable depth of those produce by cryoablation. Nevertheless, radiofrequency energy ablation resulted in a highly significantly greater area and a nearly significantly larger volume when compared to cryolesions. Moreover, colder temperatures were associated with deeper lesions and greater area and volume were associated with use of 9 F as compared to 7 F catheters. It is not clear why cryothermal energy produces a lesion with similar depth, but smaller area and volume, when compared to the one produced by a “similar” radiofrequency energy application. One plausible explanation could be the “cryoadherence” effect, which is a tight adherence of the catheter tip to the adjacent tissue caused by cooling. Due to this effect, the lesion produced is very focal, since the “brushing” of the tip electrode, usually observed during radiofrequency ablation, is missing. On the other hand, the advantage of this effect is two-fold. First, below the critical temperature of -20°C it allows good tip electrode to tissue contact, which persists throughout the whole application and it is not dependent on the torsion/deflection manoeuvres applied to the catheter. It is well known that a fixed and stable contact during the whole application is essential, especially for ablation in proximity of critical areas, such as atrioventricular node and His bundle. Second, the cryoadherence effect allows safe continuation of the application, even when sudden changes in heart rhythm that usually displace the ablation catheter (such as tachycardia termination or pacing) occur. Moreover, cryoadherence does not compromise safety, since, upon discontinuation of cryothermal energy delivery, the defrost phase is very fast (within 3 s) and the catheter can be immediately disengaged from the ablation position.

Finally, another peculiarity of cryothermal energy is the complete absence of patient symptoms in almost every case, in spite long-lasting applications. In our experience, we have evaluated patient perceptions during cryothermal energy application in a series of non-sedated cases. In almost all cases, the absolute absence of perception was demonstrated by the fact that the patient was unable to tell when the application was started and discontinued. Only in some cases, when multiple and prolonged applications are delivered in the left heart, a light sense of cold or headache is perceived as minor discomfort. Although the full explanation of the absence of symptoms is not completely clear, this characteristic can be particularly useful in young as well as in paediatric patients.

## Clinical use in ablation of cardiac arrhythmias

Over the last five yeas, the world-wide experience in catheter ablation of cardiac arrhythmias by using cryothermal energy has increased unabated. Based on this experience, cryoablation should not be viewed as a replacement for radiofrequency energy, which will continue to be the method of choice in many clinical situations. Nevertheless, ablation by cryothermal energy should be rather considered as a useful addition to the electrophysiologist’s armamentarium. In fact, different types of arrhythmias are now successfully treated by cryoablation and in some cases, especially in proximity to normal conduction pathways, treatment by this energy source is considered the first choice therapy for its safety and efficacy. The following is a brief analysis of the experience in cryoablation for each of the considered arrhythmias.

### Atrioventricular nodal reentrant tachycardia

**Figure 2A-B:**
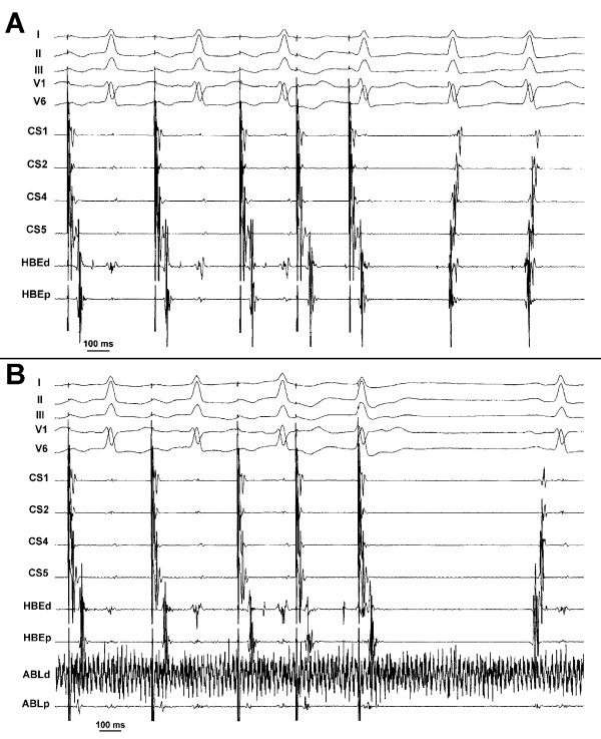
Example of suppression of inducibility of atrioventricular nodal reentrant tachycardia during cooling of the slow pathway. Form top to bottom, lead I, II, III, V1, V6, bipolar recordings from the coronary sinus catheter (from distal to proximal; CS4 is used for stimulation and not visualized) and from the distal (HBEd) and proximal (HBEp) electrode pairs of the the His bundle catheter are displayed. In panel A, programmed atrial stimulation (p=400, 260/240) from the coronary sinus reproducibly induces typical sustained atrioventricular nodal reentrant tachycardia. In panel B, also bipolar recordings from the distal (ABLd) and the proximal (ABLp) electrode pairs of the cryoablation catheter are displayed. Now, during cooling at -30°C on the slow pathway, S3 beat is blocked even at a longer coupling interval (S3= 290 ms) and tachycardia is no longer inducible. Artefacts in the ABLd are due to ice formation on the tip electrode.

So far, slow pathway ablation for atrioventricular nodal reentrant tachycardia by cryothermal energy represents the numerically larger experience in the clinical application of this new technology. Unlike radiofrequency energy, accelerated junctional rhythm is not observed during cryothermal energy application on the slow atrioventricular node pathway. Therefore, the only marker of effective ablation is suppression of tachycardia inducibility during initial cooling (Figure 2A-B). Accordingly, baseline non inducibility of the arrhythmia may be a limitation to the application of this technique. From the early report [16], several papers have contributed to accumulating experience in slow pathway ablation, with a satisfactory success rate and a recurrence rate varying from 6 to 9.7% [17-22]. In the “Frosty” trial [19], a multicentric prospective trial performed in the United States, 103 patients with atrioventricular nodal reentrant tachycardia were enrolled. On an intention-to-treat basis, the acute procedural success was 91% with no device-related complications and a recurrence rate of 6% in a 6 month follow-up. Cryomapping proved useful to predict the site of successful ablation. Nine patients had inadvertent modifications of the conduction over normal atrioventricular conduction pathways, including first to third degree atrioventricular block and right bundle branch block. These all resolved completely, usually within a minute or less and had no sequelae. A database gathering the worldwide experience and based on a combination of registry and prospective trial data reports no case of permanent atrioventricular block following cryothermal ablation of the slow pathway in more than 300 patients with atrioventricular nodal reentrant tachycardia [20]. Temporary first degree or higher atrioventricular block, observed in 15 cases (4.3%) during cryomapping at -30°C or during cryoablation at -75°C, was always reversible. Recently, the results of the first two prospective randomized trials on transvenous cryoablation versus radiofrequency ablation of the slow pathway for treatment of atrioventricolar nodal reentrant tachycardia have been published [21,22]. In these studies, cryoablation proved as effective and safe for the cure of atrioventricular nodal reentrant tachycardia as radiofrequency ablation. The higher recurrence rate that may be observed in the cryoablation group [21] suggests that, unlike radiofrequency ablation, prolonged energy applications and postablation waiting time are necessary when cryothermal ablation is used to minimize recurrence in the follow-up. In our own experience, we treat atrioventricular nodal reentrant tachycardia by slow pathway cryoablation in patients with particular anatomic characteristics, refractory to standard radiofrequency energy ablation or in pediatrics. Especially in cases with difficult anatomy, such as a small or distorted Koch’s triangle, the characteristics of cryothermal energy allow test of the ablation effect in particularly risky sites without producing irreversible damage to atrioventricular conduction, if the application is timely interrupted. In some complex cases, we found it necessary to resort to longer cryothermal energy applications (up to 480 s) and to prolong postablation observation up to 60 min. In a patient with atrioventricular nodal reentrant tachycardia, who underwent multiple unsuccessful ablation of the slow pathway, we decided to target the fast pathway by cryothermal energy [23]. In this particular case, selective and safe ablation of the fast pathway at the apex of the Koch’s triangle was accomplished and this resulted in permanent cure of the arrhythmia.

According to the presented data, cryothermal energy is a valuable and useful alternative to radiofrequency energy to treat patients with atrioventricular nodal reentrant tachycardia. Absence of permanent inadvertent damage of atrioventricular conduction makes this new technology particularly useful in cases with difficult anatomy, unsuccessful prior standard ablation procedure, in pediatrics and in all cases, in whom even the lower risk of atrioventricular block still possible with radiofrequency energy, is considered unacceptable.

### Accessory pathways

**Figure 3:**
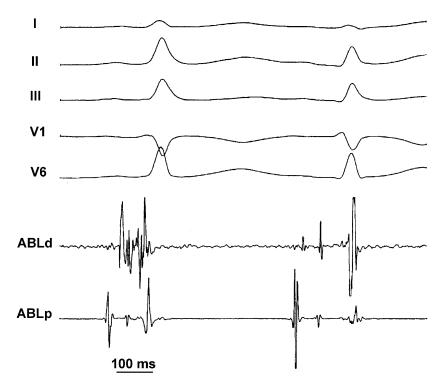
Example of disappearance of ventricular preexcitation in a case of parahissian accessory pathway. Surface ECG and bipolar recordings from the distal (ABLd) and proximal (ABLp) electrode pairs of the cryoablation catheter are displayed. The relative position of His bundle and the accessory pathway has been identified during accurate mapping, also during orthodromic atrioventricular reentrant tachycardia. Accessory pathway turned out to be located at the same site where a high amplitude His bundle potential was recorded. In this figure, the tip electrode temperature is -23°C and minor artefacts in ABLd suggest that ice is forming on the tip electrode. In the first sinus beat, ventricular preexcitation is still present with optimal A-V and V-delta interval recorded at this site. In the second beat, conduction over the accessory pathway is interrupted with disappearance of ventricular  preexcitation; now a high amplitude His bundle potential is well evident in the distal electrode pair of the ablation catheter. Permanent ablation of this parahissian pathway could be accomplished by limited cryothermal energy delivery in this site with no modification of conduction over the normal atrioventricular conduction pathway.

In Table 1, published data on cryothermal ablation of anteroseptal (parahissian) and midseptal accessory pathways are reported [17,19,24-29]. As shown, this technique in anterospetal and midseptal areas, both at high risk of complete permanent atrioventricular block when standard radiofrequency energy in performed, is highly safe and successful. In the larger series, success rate is above 90%. Although transient modifications of the normal atrioventricular nodal conduction pathways are observed during cooling, no permanent modifications is observed with the only exception of right bundle branch block in 2 cases in a single centre. In fact, immediate discontinuation of cryothermal energy application at any temperature upon observation of modification of conduction over normal pathways results in return to baseline condition, soon after discontinuation. Resumption of accessory pathway conduction with palpitation recurrences may occur in the follow up to 20%, but, especially in young healthy individual, a recurrence is by far more acceptable than permanent complete atrioventricular block requiring pacing, which was invariably the case in many series of radiofrequency ablation of these pathways. In our experience, we have treated 18 patients with anteroseptal or midseptal accessory pathways, so far, age ranging 11-51 years. No patient was excluded from the study for proximity of the accessory pathway to the normal conduction pathways (Figure 3). Successful ablation was obtained in all, but 1 pediatric and asymptomatic patient, in whom conduction properties over the accessory pathway indicated ablation, which was eventually postponed. Cryoadherence effect proved very useful in every case, but especially when energy delivery was performed during orthodromic atrioventricular tachycardia, to better visualize the His bundle electrogram and to monitor conduction over normal pathways. No complication or palpitation recurrences were observed during a 17±10 month follow-up. In approaching anteroseptal and midseptal accessory pathways, instead of performing “cryomapping” at -30°C in the selected site, we found it useful to test cryothermal energy applications with a step-by-step method to decrease temperature. In fact, in the most suitable site with the best contact (sometimes, a superior vena cava approach via a subclavian or a brachial vein is useful to stabilize contact), test applications are applied for 30 s, initially with a temperature of -30°C. If this test application is successful with no modification of normal conduction, then transition to ablation at -75°C up to 480 s is made. If the test application is unsuccessful, after re-warming, further 30s applications are tested, decreasing for each application the temperature by 10°C every step, up to the last application at -70°C. This is because we observed that the amount of cryothermal energy required for permanent ablation is quite individual (ranging from an application of -40°C for 40s to an application of -75°C for 480 s) and limiting test applications to only -30°C may limit the applicability of cryoablation in these patients. On the other hand, the use of cryothermal energy at temperatures lower than -30°C should be considered safer than radiofrequency energy in these critical sites.

Cryoablation can be also successfully and safely used to ablate selected cases of epicardial left-sided accessory pathways within the coronary sinus, well beyond the middle cardiac vein, once attempts by using both transseptal and transaortic approach have failed [20] [30]. Similarly, safe and successful cryothermal energy ablation of permanent junctional reciprocating tachycardia has been reported in children, in the midseptal region, at the coronary sinus os or in the middle cardiac vein [31].

The experience of cryoablation in unselected accessory pathways is more limited and less satisfactory [20]. Of 51 accessory pathways with various locations, only 69% were successfully ablated and this value is considerably lower than the one reported for radiofrequency ablation. There are many possible explanations for this including the learning curve and the smaller size of the lesion produced by cryoablation [10]. In any case, all the peculiarities of cryothermal energy, which are optimal for septal ablation, are less important or even useless for ablation of accessory pathways located elsewhere.

### Focal atrial tachycardia and isthmus-dependent atrial flutter

Occasionally, successful cryoablation of focal atrial tachycardia has been reported and its safety has been confirmed also for ablation of atrial foci located close to the atrioventricular node [32].

Several papers have reported cryoablation of the cavotricuspid isthmus for typical atrial flutter with an acute and long-term success comparable to the one of radiofrequency ablation [33-36]. The use of larger catheter and longer electrode for ablation in this area is associated with a lower number of applications and a shorter procedural time. As for radiofrequency ablation, a case of transient ST segment elevation in the inferior leads was observed during cryo application at the septal isthmus, with wall irregularities in the right coronary artery without significant stenosis [35]. The major advantage of using cryothermal energy to produce bidirectional conduction block of the cavo-tricuspid isthmus is the absence of pain perception related to energy application. In a prospective randomized trial in which a visual analogue scale to evaluate pain was used, pain perception was by far lower if not existent in the cryothermal as compared to radiofrequency energy group [34].

### Pulmonary vein ablation for atrial fibrillation

When radiofrequency energy is applied at the os of the pulmonary veins to prevent atrial fibrillation recurrences, a heat-induced contraction of the pulmonary vein wall can be observed early or during the follow-up, which results in a variable degree of lumen reduction and a wide spectrum of clinical presentations [37]. This reaction is typical of hyperthermic injury and results from a combination of edema, endothelial disruption and collagen denaturation and shrinkage [38]. The occurrence and the degree of stenosis correlate with the amount of energy delivered [39] and lesion extension [40]. As mentioned above, cryothermal energy ablation causes less or minimal endothelial disruption, maintenance of extracellular collagen matrix and no collagen contracture related to thermal effects. Moreover, lower incidence of thrombus formation is reported with cryoenergy as compared to radiofrequency energy ablation. For these characteristics, cryothermal energy ablation can be considered an ideal and safer energy source also for pulmonary vein ablation and the incidence of both pulmonary veins stenosis and thromboembolic events is expected to be dramatically reduced by using cryoablation. On the other hand, the presence of high blood flow in the pulmonary vein may represent a considerable heat load, which may limit the size and depth of the lesion produced by cryothermal energy at the os of the pulmonary vein. Moreover, the longer time required to produce a permanent lesion may relevantly reflect on procedure duration, limiting the clinical use of this theoretically optimal energy source. Initial experiences of electrophysiologically-guided segmental ostial ablation of the pulmonary vein by using cryothermal energy application with 10 or 7 F catheters [20,41] have been reported. These experiences show that pulmonary vein isolation is feasible with a comparable number of applications and clinical outcome with regard to radiofrequency ablation; longer procedural times, observed for both the 10 F and the 7 F catheter, correlate with longer application times required when cryothermal energy is used. Importantly, the early cryoablation experience has not evidenced, so far, development of pulmonary veins stenosis following ablation. Technologic evolution is now aimed to develop new catheter designs for circumferential ostial ablation of the pulmonary veins, with the option of deploying in the pulmonary veins an inflatable balloon to reduce the heat load related to blood flow [20]. These devices are to be tested in a large patient cohort to assess whether these technological improvements will lead to optimization of the use of cryothermal energy, maximizing the advantages of this new technology and limiting the drawbacks encountered in its clinical use.

### Ventricular arrhythmias

Although clinical data on cryothermal ablation of ventricular arrhythmias are missing, preliminary experimental evidence shows that percutaneous cryoablation in several sites of normal ventricular myocardium is feasible with lesion deeper in the left than in the right ventricle, probably due to better contact in the former than in the latter [42]. In the same study, cryothermal energy has been also tested to ablate sustained ventricular tachycardias in a post-infarction sheep model. A limited number of applications was effective in suppressing the inducibility of ventricular arrhythmias, producing a transmural lesion in the majority of the cases with no acute complication.

Interestingly, cryothermal energy could be used to target ventricular tachycardias of epicardial origin, once the epicardial space has been reached by the non surgical transpericardial approach, originally described by Sosa [43]. As compared to radiofrequency energy, cryothermal energy seems to be safer in the epicardium, due to less probable damage to epicardial coronary arteries. The reduced heat load in the pericardial space related to the absence of blood flow could be to the advantage of cryoablation in these cases, with the possibility to produce larger transmural lesions.

## Figures and Tables

**Table 1:**
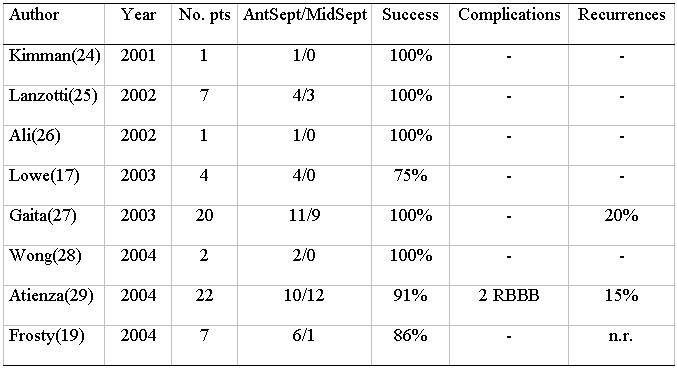
Review of cryoablation of anteroseptal or midseptal accessory pathways

Abbreviations: No.pts: number of patients; AntSept: number of patients with anteroseptal accessory pathways; MidSept: number of patients with midseptal accessory pathways; RBBB: right bundle branch block; n.r.: not reported
